# Management and Follow-up of Patients with a Bronchial Neuroendocrine Tumor in the Last Twenty Years in Ireland: Expected Inconsistencies and Unexpected Discoveries

**DOI:** 10.1155/2018/1043287

**Published:** 2018-08-29

**Authors:** Asta Agasarova, Clare Harnett, Niall Mulligan, Muhammad Shakeel Majeed, Alberto Caimo, Gianluca Tamagno

**Affiliations:** ^1^Department of Endocrinology/Diabetes Mellitus, Mater Misericordiae University Hospital, Dublin, Ireland; ^2^Department of Pathology, Mater Misericordiae University Hospital, Dublin, Ireland; ^3^School of Mathematical Sciences, Dublin Institute of Technology, Dublin, Ireland

## Abstract

Bronchial neuroendocrine tumors (NET) are classified into well-differentiated typical carcinoids (TC), atypical carcinoids (AC), large cell neuroendocrine carcinomas (LCNEC), and small cell lung carcinomas (SCLC). We retrospectively reviewed and analyzed the diagnostic and therapeutic aspects, follow-up data, and outcomes of all patients diagnosed with a bronchial NET from 1995 to 2015 at our institution. Patients with LCNEC or SCLC were excluded due to the biological and clinical differences from the other bronchial NET. The clinical, laboratory, imaging, treatment, and follow-up data were collected and analyzed keeping in mind the recently published international recommendations. Forty-six patients were included in the study. Of these, 37 had a TC and 5 an AC. In 4 patients, the histological characterization was inadequate. Forty-four patients underwent surgery. Four patients developed metastatic disease. Interestingly, 14 patients had one or more other tumors diagnosed at some stage and 3 of them had three different tumors. A total of 7 patients died. The analysis of the laboratory and pathology assessment identified some inconsistencies when compared to the international recommendations. Although the treatment of bronchial NET at our institution was consistent with the successively published recommendations, it appears that the diagnostic process and the follow-up surveillance were not. We think that a systematic multidisciplinary approach might improve bronchial NET patient care. A relatively high rate of occurrence of a second, or also a third, non-NET tumor was observed, though the statistical value of such observation could not be exhaustively elucidated in this numerically limited patient population. In our opinion, the observed high rate of second malignancies in this patient cohort highlights the necessity of optimizing the follow-up of the bronchial NET patients, also considering the very good survival rate achieved with regard to the bronchial NET.

## 1. Introduction

Bronchial neuroendocrine tumors (NET) belong to the heterogeneous group of rare neoplasms originating from the enterochromaffin cells which are diffusely distributed in the body [1]. The incidence rate of bronchial carcinoids ranges from 0.2 to 2/100,000 population per year in both US and European countries [2]. The incidence of these tumors has increased significantly over the past 30 years, at a rate of approximately 6% per year, due to improved awareness and available diagnostics [2]. The World Health Organization (WHO) identifies four histological variants of bronchial NET, namely, well-differentiated typical carcinoids (TC), atypical carcinoids (AC), large cell neuroendocrine carcinomas (LCNEC), and small cell lung carcinomas (SCLC) [3]. The most relevant aspect of the 2015 WHO classification in comparison to the previous classification is represented by the separation of carcinoids (either typical or atypical) from LCNEC and SCLC, which are grouped together [3, 4]. Depending on their grade of malignancy, bronchial NET can also be classified into low grade TC, intermediate grade AC, and high grade LCNEC/SCLC [3]. TC usually grow slowly and do not often spread beyond the lungs. AC grow faster than the typical tumors and are more likely to metastasize to other organs. LCNEC, a rare form of poorly differentiated malignant tumor, are similar to SCLC in terms of prognosis and treatment. SCLC represent one of the most rapidly growing types of cancer and determine a poor prognosis [5]. SCLC are NET from a biological point of view but their behavior is usually very aggressive and, conversely, the management of these lesions also differs very much from the other bronchial NET [6]. SCLC and LCNEC are associated with heavy smoking and recent reports confirm the role of smoking in AC development as well [7, 8]. TC and AC are usually sporadic tumors; however, rare familial cases have been reported [9]. They may be associated with multiple endocrine neoplasia type 1 in less than 5% of patients [10]. The occurrence of carcinoid syndrome or symptoms of Cushing's syndrome are rare and occurrence of tumor-associated acromegaly is pretty much anecdotal [2]. The clinical picture may include symptoms such as cough, haemoptysis, dyspnoea, chest pain, or wheezing, or could be even completely asymptomatic [11].

Computed tomography (CT) scan with contrast is the gold standard for bronchial NET diagnostic imaging, but pathological examination is mandatory for their correct classification [2]. Ki-67 proliferation index helps to distinguish well-differentiated tumors from the poorly differentiated ones [5]. The major pathological biomarkers are chromogranin A, synaptophysin, and CD56 [12]. Flexible bronchoscopy is indicated in all the central forms of bronchial NET, while rigid bronchoscopy may be preferred in patients at high risk for bleeding [2]. Nuclear medicine techniques (octreoscan, gallium-68 DOTA positron emission tomography) are more specific than conventional imaging, enable whole-body imaging for staging, and may help to predict the response to peptide receptor radionuclide therapy (PRRT), if necessary at any stage of the therapeutic management [11].

Surgical resection is the treatment of choice for bronchial NET [1, 2, 13, 14]. In cases with advanced disease, cytoablative procedures such as locoregional therapy and PRRT may represent effective palliative therapeutic options [15]. Cytotoxic treatment has been the standard for aggressive bronchial NET, although the available chemotherapy regimens demonstrate a limited effect [2]. The inconsistencies between the recommendations for systemic therapies, especially radiation and chemotherapy, result in a lack of consensus on a standardized treatment for unresectable disease [16]. Somatostatin analogs are considered as first-line treatment for patients with advanced unresectable bronchial NET with low proliferation index and positive octreoscan. Two large studies (PROMID and CLARINET) have demonstrated an improved progression-free survival in patients with a digestive tract NET treated with somatostatin analogs [17, 18]. However, there is no extensive and conclusive data on the anti-proliferative effects of somatostatin analogs in bronchial NET to date. Indeed, the knowledge and expertise regarding bronchial NET internationally has developed much more slowly than that of NET of the digestive tract in the last decade and this issue highlights the need to focus more extensively on this area with the ultimate goal of improving patient care.

In this retrospective study, we have analyzed the diagnostic and therapeutic approach to the patients with TC and AC in our institution in the last 20 years and compared them with the recommendations recently published for assessing the quality of the clinical service provided so far. In the last twenty years, a structured care for the management of bronchial NET patients including a dedicated multidisciplinary approach did not exist at our institution. Moreover, we have assessed the clinical outcomes on the basis of patient records and recorded the most relevant findings.

## 2. Patients and Methods

One hundred and seven patients with a bronchial NET have been diagnosed, treated, or followed-up at our institution from 1^st^ January 1995 to 31^st^ December 2015. Sixty patients have been excluded as they had SCLC and one patient has been excluded as she had a LCNEC. We excluded the patients with LCNEC or SCLC due to the biological and clinical differences between these tumors and the other bronchial NET. No other exclusion criteria applied. Thus, forty-six patients with TC or AC have been finally included in our study. The most relevant anthropometric data of the patients and the general information regarding their inpatient or outpatient care at our institution were recorded in an electronic database, built for the scope. We have collected laboratory, imaging, treatment, and follow-up data from patient charts and the electronic system of the hospital. We analyzed these findings and compared them with the international recommendations published in 2015 [2]. Moreover, we have divided the patients in chronological subgroups and analyzed the differences in terms of professional practice. The aim was to assess the management of patients with a bronchial NET in the last twenty years and identify the weakest areas in the management of these patients with the ultimate goal of improving future patient care. The blood tests were performed in the laboratory of our institution, with the exception of chromogranin A that was sent abroad (Laboratory of Neuroendocrinology, Royal Victoria Hospital, Belfast, UK). The majority of the radiological investigations were done at our institution. When the patient had imaging done elsewhere, we collected and reviewed the scans at the radiology department of our institution. The pathology specimens have been processed and analyzed at our institution and if the specimen was collected elsewhere, the pathology block was subsequently processed and reviewed at our institution.

With regard to the chronological subgroup analysis of the professional practice, we have applied chi-squared tests for testing the differences between the periods with a statistically sufficient number of patients. Since a quite large number of other tumors appeared in our patient population, a Bayesian Fisher exact test on contingency tables was performed to investigate the proportion of occurrences of the second and third tumors, and by using simple uniform priors on the proportions, the posterior distribution of the beta-binomial model was assessed. Unfortunately, the comparison of the data on the occurrences of the second and third tumors with the corresponding data from the general oncological population in Ireland was not possible due to the unavailability of a national database record.

This study is compliant with the declaration of Helsinki of the World Medical Association and as per its audit nature did not require ethical committee approval.

## 3. Results

Forty-six consecutive patients were collected. Twenty-three patients were female (48.9%), and twenty-four were male (51.1%). The mean age was 55 years (range: 15–82 years). Thirty-seven patients had a histological diagnosis consistent with TC (80.4%) and five with AC (10.9%) (Table 1). Four patients were diagnosed with a bronchial NET but the histological characterization of their specimens was inadequate for a more precise definition (8.7%).

Four patients with a bronchial NET had metastatic disease at some stage (8.7%). Three of them had evidence of metastases already at the time of the diagnosis of bronchial NET. Two of them had lymph node metastases and underwent lymphadenectomy, two patients had liver involvement (both non-treated due to poor prognosis), and one of them later developed lung recurrence of the NET. The patient with liver metastases and lung reoccurrence of the tumor was apparently free from metastatic disease at the time of the bronchial NET diagnosis (AC).

Chest X-ray, chest CT, and bronchoscopy before surgery (or decision not to operate) were performed in 100% of the patients. Specimens for histological examination were available for all patients. During the follow-up, chest X-ray was done in 93.5% and CT in 65.2% of the patients. However, these investigations have not been carried in a timely fashion according to the international recommendations but were performed mostly in a random way, without the identification of a defined follow-up pattern. On the contrary, a lack of consistency in the laboratory (chromogranin A never assessed) and pathology (Ki-67 assessed in 34.8%, mitotic count in 58.7%, and chromogranin A in 54.3% of the patients, synaptophysin in 60.9% of the patients) findings before/at the time of surgery as well as in the laboratory and endoscopic assessment during the follow-up (chromogranin A assessed in 2.2% and bronchoscopy performed in 8.7% of the patients) was identified (Table 2). At histology, chromogranin A was positive in 20 patients (80.0%) and negative in five patients (20.0%), while synaptophysin was positive in every specimen tested for. Other histological markers have been randomly checked (CD56 — *n* = 4, cytokeratins AE1/3 — *n* = 9, Leu-7 — *n* = 2, CAM — *n* = 1, and CK7 — *n* = 1) and were positive in every specimen tested for. ^18^F-FDG positron emission tomography (^18^F-FDG PET) was carried out in eighteen patients (39.1%) and was positive in seventeen patients and negative in one of them. All eighteen patients underwent pre-operative ^18^F-FDG PET and nobody underwent a post-operative ^18^F-FDG PET. Octreoscan was carried out in six patients only (13.0%). Three of them had a somatostatin receptor-positive tumor. None of the patients who had pre-operative octreoscan underwent also a post-operative octreoscan.

Forty-four patients (95.6%) underwent surgery. One of the patients was not treated as she was unfit for surgery or systemic treatments because of her poor lung function and did not receive any treatment (Table 3). One patient with synchronous squamous cell carcinoma of the lung was treated with both chemotherapy (5-fluorouracil with temozolomide) and radiotherapy.

The analysis of the professional practice among the chronological subgroup of patients was possible only between the chronological groups 2006–2010 and 2011–2015 (Tables 4, 5, and 6). There is no evidence to suggest any difference between these two time frames (*p*-value = 0.6394). We could not compare the other time frames due to the insufficient number of patients (<5) in each subgroup.

At the time of the data collection, thirty-eight patients were alive (82.6%) and seven patients had died (15.2%), among which three died because of recurrence and/or metastases of bronchial NET. Four patients died as a consequence of other clinical issues (colorectal cancer, stroke). The outcome of one patient is unknown.

**Figure 1:**
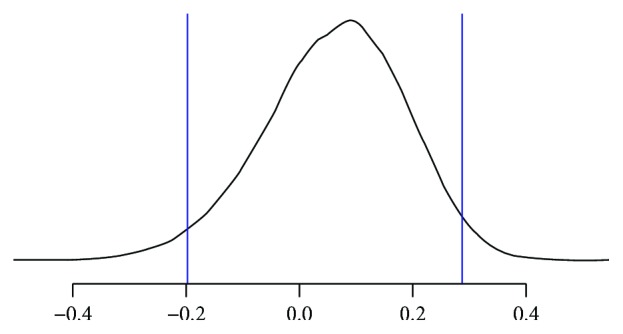
Posterior probability distribution of the mean difference between the proportion of occurrence of a second tumor and the proportion of occurrence of a third tumor in our patient population. The vertical lines are drawn at the boundaries of the central 95% region of the posterior difference.

Fourteen patients (30.4%) also had one or more other tumors diagnosed during the observation time and three of them had a total of three tumors identified (Table 7). The first of these three patients had TC, right renal cancer, and benign cystic parotid tumor. The second patient had AC, adenocarcinoma of the lung, and colorectal cancer. In the third case the patient had TC, melanoma, and a basal cell carcinoma. Five patients (29.4%) were diagnosed with another tumor before the diagnosis of the bronchial NET (over six months), and eight patients (47.1%) had synchronous tumors (within 6 months before or after the diagnosis of bronchial NET). Four patients (23.5%) had metachronous tumors (diagnosed over 6 months from the diagnosis of bronchial NET) (Table 8). Among the non-NET tumors, we had found one squamous cell carcinoma excised from the cheek, two colorectal cancers (the first tumor surgically resected and the second tumor resected and treated with adjuvant chemotherapy), one metastatic squamous cell carcinoma of unknown primary treated with radical axillary lymphadenectomy and subsequent adjuvant radiotherapy, three lung squamous cell carcinomas (one treated with upper lobectomy and adjuvant chemotherapy, one with upper lobectomy, and one with chemo-radiotherapy), two renal cancers which underwent nephrectomy, a benign cystic tumor of the parotid which was removed surgically, a lung adenocarcinoma treated with lower lobectomy, a papillary thyroid cancer treated with thyroidectomy, an atrial myxoma which was resected, a breast cancer treated surgically, an ovarian cancer treated with chemo-radiotherapy, a melanoma which was resected, and a basal cell carcinoma excised from the pre-auricular area. As per our statistical model based on the observed data, there is a 95% probability that the difference between the proportion of second tumor occurrence and the proportion of third tumor occurrence falls in the range between −0.283 and 0.287, meaning that there is no significant difference between the two proportions (Figure 1).

## 4. Discussion

The scientific knowledge regarding bronchial NET has increased more slowly than that of NETs of the digestive tract over the last couple of decades. Only recently have expert recommendations for the diagnosis and management of bronchial NET been published [2]. It appears that both scientists and physicians dealing with these tumors should aim to increase awareness in the field and improve the management of patients with bronchial NET. In this retrospective study, we have analyzed the diagnostic and therapeutic approach to patients with a bronchial NET in our institution in the last 20 years and compared the findings with the recently published international recommendations.

From a diagnostic point of view, all patients with bronchial NET should have plasma chromogranin A levels assessed, which never happened in our patient series. At histopathology, Ki-67 and chromogranin A should be assessed in all specimens; however, in our series these markers were checked only in 34.8% and 54.3%, respectively. From a therapeutic point of view, most patients have received the appropriate surgical management of their tumor. Another patient with synchronous lung squamous cell carcinoma was treated with both chemotherapy and radiotherapy and the main goal was the treatment of her non-neuroendocrine neoplasm. None of the patients received somatostatin analogs. Indeed, none of them had carcinoid syndrome. It is noteworthy to report that for most of the observation time the evidence of an anti-proliferative effect of the somatostatin analogs was still uncertain, while now, at least for most of the NETs arising from the digestive tract, this is a demonstrated cornerstone. One patient in our series did not receive any treatment as she was unfit for surgery and no other form of therapy was prescribed due to her co-morbidities and poor baseline. This patient had liver metastases and her final outcome was poor. Considering the most recent evidence and the international recommendations, she may have been a candidate for treatment with somatostatin analogs. However, in general terms, the therapeutic management of bronchial NET in our institution appears to reasonably match with international guidelines. In the follow-up, conventional imaging should be carried out at 3 and 6 months and then yearly in patients with TC, in association with the measurement of circulating chromogranin A for the first 2 years. Then, annual chest X-ray and CT every 3 years are recommended. For patients with AC, closer monitoring is recommended and CT is advised at 3 months post-surgery, then, every 6 months for 5 years, and later every year. Bronchoscopy should be carried out on a routine basis every 5–10 years for TC and 1–3 years for AC. In our patient series, follow-up bronchoscopy was performed in four patients (8.7%). Three of these patients had TC and underwent bronchoscopy in one, five, and seven years, respectively. One patient with inadequate histological characterization of the specimen underwent bronchoscopy in one year and did not undergo any further bronchoscopy following this. During the follow-ups, chest X-ray was done in 93.5% and chest CT in 65.2% of the patients, but these investigations have been carried out randomly in terms of timing, without evidence of a specific follow-up strategy. In the laboratory follow-up, chromogranin A was assessed in only one patient and for once only. These findings suggest that the follow-up of bronchial NET patients at our institution has been mostly inconsistent with the current international recommendations [2]. However, the relevance of follow-up in bronchial NET patients is a matter of debate. Recently, a retrospective study tried to determine the relevance of close post-resection surveillance for bronchopulmonary carcinoids. Fifty-seven patients underwent lung resection between 2006 and 2013, most of them for TC (93%). Eighteen patients underwent post-operative bronchoscopy surveillance without showing any recurrence of the disease. A total of 146 follow-up CT scans were performed on fifty-three patients and, again, no disease recurrence was detected. As per the authors, on the base of their findings, close surveillance following complete resection of a TC is probably unnecessary [19].

The retrospective analysis of the diagnostic process and follow-up of this cohort of patients in our institution in the last twenty years showed a partial lack of consistency in the laboratory and pathology assessment before treatment and some inconsistencies in the laboratory and imaging findings/endoscopic assessment during follow-up. It appears that the main issue was represented by the timing of the restaging during the follow-up, together with the performance of a complete assessment including an exhaustive histological characterization. Possibly, the diagnostic and follow-up inconsistencies could be reduced by a multidisciplinary approach [20, 21]. In concordance with the recommendations, the management of bronchial NET should be shared by the multidisciplinary team (MDT) including oncologists, radiologists, pathologists, surgeons, and internists (i.e., respiratory physicians and endocrinologists). In 2013, a study focusing on the impact of a systematic multidisciplinary approach on the management of patients with gastroenteropancreatic (GEP) NET illustrated for the first time that the MDT approach noticeably altered the diagnosis, the management, and the follow-up of patients with GEP NET and moved the current practice at that institution to the best international practice in the short-term (18 months) [20]. In our opinion, a multidisciplinary approach would lead to a similarly significant improvement of the care also with regard to patients with bronchial NET.

In the analysis of the patient subgroups divided per time frames, we have not reported any statistical difference regarding the diagnostic or therapeutic management of the bronchial NET patients. Probably this is a consequence of the limited number of patients, at least in part. Moreover, since no guidance was available in the literature before 2015, we think that the bronchial NET patients were simply managed following the personal expertise and judgment of the caregiver physicians, with a subsequent homogenization of the professional practice. In other countries with more developed NET services over the last two decades, the management consistently evolved overtime, also as a consequence of the revisions of the WHO classification of bronchial NETs [22, 23].

Bronchial NET may develop in patients with a previous history of cancer, especially of the respiratory tract, urogenital tract, or the skin [24]. Moreover, a second neoplasia may occur in patients with a history of bronchial NET. As reported in our case series, fourteen patients also had other tumors, corresponding to the impressive rate of almost one every three patients, and three of these patients had a total of three tumors during their life. We defined the tumors occurring within 6 months of the diagnosis of their bronchial NET as synchronous tumors. The tumors occurring more than 6 months after the diagnosis of bronchial NET have been defined as metachronous [25]. A small number of patients in our series had a non-NET neoplasia diagnosed before the diagnosis of bronchial NET. The Irish national statistics show that 30,000 new cases of cancer are diagnosed on average each year and this number is expected to rise to over 40,000 per year by 2020 [26]. A recent Italian epidemiological study analyzed 3205 patients with bronchial carcinoid in the period 1975–2011 and found a high frequency of second tumors. In particular, synchronous thyroid tumors in females and metachronous renal tumors and synchronous bladder tumors in males were reported [27]. A retrospective American study assessed the risk of second malignancies in children and adolescents with a NET in the period 1945–2012 and did not detect second primary malignancies during the routine long-term follow-up of these patients [28]. However, the large majority of these patients had an appendiceal NET and only a very few had a bronchial carcinoid. In the USA, a study on the risk of a second cancer among survivors from a previous cancer from 1992 to 2008 showed that nearly one in twelve patients diagnosed with a common cancer developed a second malignancy, the most common of which was lung cancer [29]. The predisposition to other tumors in patients with NET has been evaluated in the Danish Cancer Registry among more than one thousand patients [30]. The overall relative risk was not increased, but there was an excess of some tumor types. In particular, thyroid cancer, brain tumors, and non-Hodgkin's lymphoma were recorded more often than expected in the NET patient population. However, these findings were affected by a wide confidence interval related to the relatively small number of patients. There is data on second cancers in patients with NET also from Sweden [31], where the risk of synchronous tumors in the NET patient population was increased. However, in this study the timeframe of synchronicity was extended to 12 months. These neoplasms are mostly gastrointestinal cancers, but also the incidence of second primaries in the lung, the prostate, the kidney, and the endocrine system was increased.

The reason of the increased incidence of a second tumor has not been fully elucidated yet. Of course, this may represent an incidental diagnosis during diagnostic or follow-up investigations. Moreover, with a regular, ongoing follow-up of the bronchial NET patients in a tertiary referral center, it is likely that also standard cancer screening was carried out in a more regular way than for the general population. Finally, we cannot exclude the presence of proto-oncogene mutations which may predispose the bronchial NET patients to an additional oncological risk, though at the moment there are no literature data supporting such hypothesis. However, such findings support the relevance of an appropriate follow-up of the patients with bronchial NET because they may have a higher risk of development of second tumors. We think that the relatively high rate of second malignancies in our bronchial NET patient cohort emphasizes the need of an optimization of the follow-up of the bronchial NET patients, especially if we keep in mind the well-known findings of good survival reported for the majority of the patients with a bronchial NET. Moreover, it appears that large and homogeneous international epidemiological studies addressing the risk of second neoplasms in NET patients, and in particular in bronchial carcinoid patients, are required based on the suspicious though non-conclusive findings so far.

## 5. Conclusions

Although the therapeutic management of bronchial NET in our institution appears to match reasonably with the international recommendations, the diagnostic process and the follow-up of these patients was not consistent. We think that the optimization of care for patients with bronchial NET is an absolute requirement at this stage and should mirror the evolution of the care of GEP NET patients in the last two decades. A systematic multidisciplinary approach would likely improve the still inconsistent aspects of care of patients with bronchial NET with the ultimate goal of improving their clinical outcomes. Impressively, almost one in every three bronchial NET patients in our series also had a second tumor at some stage. Keeping together our findings and the literature, it appears that a careful follow-up of bronchial NET patients is also required considering the emerging evidence of a possible high risk of development of second malignancies in this patient group, though larger and dedicated international epidemiological studies are necessary for confirming the suspicion rising from our retrospective study.

## Figures and Tables

**Table 1:** Characteristics of bronchial NET patients diagnosed, treated, or followed-up at our institution from 1^st^ January 1995 to 31^st^ December 2015 (*n* = 46).

*Age at the time of diagnosis* (mean ± SD and range)	55.2 ± 16.0 years
	15–82 years
*Gender* (*n* and % females, *n* and % males)	22 females (47.8%)
	24 males (52.2%)
*Tumor type* (*n* and %)	
Typical	37 (80.4%)
Atypical	5 (10.9%)
Unknown	4 (8.7%)
*Metastatic disease* (*n* and %)	4 (8.7%)

*n*: number of patients, SD: standard deviation.

**Table 2:** Biochemistry, imaging, bronchoscopy, and pathology findings in bronchial NET patients diagnosed, treated, or followed-up at our institution from 1^st^ January 1995 to 31^st^ December 2015 (*n* = 46). Treatment refers to surgery, chemotherapy, or radiotherapy.

Parameter	Number of patients (%)
*Chromogranin A* (plasma)	Pre-treatment 0 (0%)
	Post-treatment 1 (2.2%)
*Ki-67* (histology)	16 (34.8%)
*Mitotic count* (histology)	27 (58.7%)
*Synaptophysin* (histology)	28 (60.9%)
*Chromogranin A* (histology)	25 (54.3%)
*CD56* (histology)	11 (23.9%)
*Bronchoscopy*	Pre-treatment 46 (100%)
	Post-treatment 4 (8.7%)
*CT*	Pre-treatment 46 (100%)
	Post-treatment 30 (65.2%)
*Chest X-ray*	Pre-treatment 46 (100%)
	Post-treatment 43 (93.5%)
*^18^F-FDG PET*	Pre-treatment 18 (39.1%)
	Post-treatment 0 (0%)
*Octreoscan*	Pre-treatment 4 (8.7%)
	Post-treatment 2 (4.3%)

*n*: number of patients.

**Table 3:** Therapeutic management of bronchial NET patients diagnosed, treated, or followed-up at our institution from 1^st^ January 1995 to 31^st^ December 2015 (*n* = 46). One patient underwent both radiotherapy and chemotherapy.

Treatment	Number of patients (%)
*Surgery*	44 (95.6%)
*Somatostatin analogs*	0 (0%)
*Interferon alpha*	0 (0%)
*Peptide receptor radionuclide therapy*	0 (0%)
*Radiotherapy*	1 (2.2%)
*Chemotherapy*	1 (2.2%)
*Locoregional therapy of distant metastases (radiofrequency ablation, chemoembolization)*	0 (0%)
*No treatment*	1 (2.2%)

*n*: number of patients.

**Table 4:** Characteristics of bronchial NET patients managed at our institution divided in chronological time frames.

	1995–2000	2001–2005	2006–2010	2011–2015
	*n* = 5	*n* = 2	*n* = 20	*n* = 19
*Age at the time of diagnosis* (mean ± SD and range)	57.6 ± 25.4	40.5 ± 36.1	54.7 ± 13.8	56.7 ± 14.1
	15–82	15–66	27–76	21–73
*Gender* (*n* and % females, *n* and % males)	3 (60.0%)	1 (50.0%)	6 (30.0%)	12 (63.2%)
	2 (40.0%)	1 (50.0%)	14 (70.0%)	7 (36.8%)
*Tumor type* (*n* and %)				
Typical	3 (60.0%)	1 (50.0%)	18 (90.0%)	15 (78.9%)
Atypical	1 (20.0%)	0 (0%)	1 (5.0%)	3 (15.8%)
Unknown	1 (20.0%)	1 (50.0%)	1 (5.0%)	1 (5.3%)
*Metastatic disease* (*n* and %)	1 (20%)	0 (0%)	1 (5.0%)	2 (10.5%)

*n*: number of patients, SD: standard deviation.

**Table 5:** Biochemistry, imaging, bronchoscopy, and pathology findings in bronchial NET patients managed at our institution divided in chronological time frames.

	1995–2000	2001–2005	2006–2010	2011–2015
	*n* = 5	*n* = 2	*n* = 20	*n* = 19
*Chromogranin A* (plasma)	0 (0%)	0 (0%)	0 (0%)	0 (0%)
	0 (0%)	0 (0%)	1 (5.0%)	0 (0%)
*KI-67* (histology)	0 (0%)	0 (0%)	3 (15.0%)	13 (68.4%)
*Mitotic count* (histology)	2 (40.0%)	0 (0%)	12 (60.0%)	13 (68.4%)
*Synaptophysin* (histology)	3 (60.0%)	0 (0%)	12 (60.0%)	13 (68.4%)
*Chromogranin A* (histology)	3 (60.0%)	0 (0%)	8 (40.0%)	14 (73.7%)
*CD 56* (histology)	0 (0%)	0 (0%)	2 (10.0%)	9 (47.4%)
*Bronchoscopy*	5 (100%)	2 (100%)	20 (100%)	19 (100%)
	0 (0%)	0 (0%)	3 (15.0%)	1 (5.3%)
*CT*	5 (100%)	2 (100%)	20 (100%)	19 (100%)
	4 (80.0%)	1 (50.0%)	13 (65.0%)	12 (63.2%)
*Chest X-ray*	5 (100%)	2 (100%)	20 (100%)	19 (100%)
	5 (100%)	2 (100%)	19 (95.0%)	17 (89.5%)
*^18^F-FDG PET*	0 (0%)	0 (0%)	10 (50.0%)	8 (42.1%)
	0 (0%)	0 (0%)	0 (0%)	0 (0%)
*Octreoscan*	0 (0%)	0 (0%)	1 (5.0%)	3 (15.8%)
	0 (0%)	0 (0%)	2 (10.0%)	0 (0%)

*n*: number of patients.

**Table 6:** Therapeutic management of bronchial NET patients managed at our institution divided in chronological time frames.

	1995–2000	2001–2005	2006–2010	2011–2015
	*n* = 5	*n* = 2	*n* = 20	*n* = 19
*Surgery*	5 (100%)	2 (100%)	20 (95.2%)	17 (89.5%)
*Somatostatin analogs*	0 (0%)	0 (0%)	0 (0%)	0 (0%)
*Interferon alpha*	0 (0%)	0 (0%)	0 (0%)	0 (0%)
*Peptide receptor radionuclide therapy*	0 (0%)	0 (0%)	0 (0%)	0 (0%)
*Radiotherapy*	0 (0%)	0 (0%)	0 (0%)	1 (5.3%)
*Chemotherapy*	0 (0%)	0 (0%)	0 (0%)	1 (5.3%)
*Locoregional therapy of distant metastases (radiofrequency ablation, chemoembolization)*	0 (0%)	0 (0%)	0 (0%)	0 (0%)
*No treatment*	0 (0%)	0 (0%)	0 (0%)	1 (5.3%)

*n*: number of patients.

**Table 7:** Contingency table showing the number of second tumors diagnosed in patients with a bronchial NET and the number of third tumors diagnosed in patients with a bronchial NET and a second tumor.

	2^nd^ tumor	3^rd^ tumor
*Number of patients*	46	14
*Number of events (second / third tumor occurrences)*	14	3

**Table 8:** Other tumor types recorded in bronchial NET patients diagnosed, treated, or followed-up at our institution from 1^st^ January 1995 to 31^st^ December 2015 (*n* = 46).

Non-NET tumors that occurred in patients with a bronchial NET	Number of patients (and %)
*Total number of patients with other tumors*	14 (30.4%)
*Total number of other tumors*	17 (37.0%)
(i) Before the diagnosis of bronchial NET (ii) Synchronous tumors (iii) Metachronous tumors	(i) 5/17 (29.4%) (ii) 8/17 (47.1%) (iii) 4/17 (23.5%)
*Tumor types*	
(i) Cheek squamous cell carcinoma (ii) Colorectal cancer (iii) Metastatic squamous cell carcinoma of unknown primary (iv) Lung squamous cell carcinoma (v) Renal cancer (vi) Salivary gland benign cystic tumor (vii) Lung adenocarcinoma (viii) Thyroid cancer (ix) Atrial myxoma (x) Breast cancer (xi) Ovarian cancer (xii) Melanoma (xiii) Basal cell carcinoma	(i) 1 (5.9%) (ii) 2 (11.8%) (iii) 1 (5.9%) (iv) 3 (17.6%) (v) 2 (11.8%) (vi) 1 (5.9%) (vii) 1 (5.9%) (viii) 1 (5.9%) (ix) 1 (5.9%) (x) 1 (5.9%) (xi) 1 (5.9%) (xii) 1 (5.9%) (xiii) 1 (5.9%)

*n*: number of patients.

## Data Availability

The clinical data used in this study are confidential in order to protect patient privacy. The detailed data analysis to support the findings of this study is available from the corresponding author upon request.
